# The Place of Reductive Surgery in the Management of Gestational Ulcerative Gigantomastia: A Case Report at Sourô Sanou Teaching Hospital

**DOI:** 10.1155/2019/7946240

**Published:** 2019-05-20

**Authors:** Somé Ollo Roland, Zaré Cyprien, Konkobo Damien, Dembélé Adama, Konségré Valentin, Yabré Nassirou, Bambara Moussa

**Affiliations:** ^1^Department of Surgery of the CHUSS of Bobo Dioulasso, Burkina Faso; ^2^Department of Gynecology and Obstetrics of the CHUSS of Bobo Dioulasso, Burkina Faso; ^3^Department of Anatomo-Pathology of the CHUSS of Bobo Dioulasso, Burkina Faso

## Abstract

**Introduction:**

Gestational gigantomastia is a rare benign disease of unknown cause. We report a case of bilateral gigantomastia in pregnancy in order to locate the place of reductive surgery in its care which is quite controversial.

**Case:**

A 25-year-old woman gravida 2 para 1 was referred for an exaggerated bilateral breast enlargement at 32-week gestation. The examination showed bilateral giant breasts with collateral venous circulation and trophic changes marked by the necrosis of the distal third of the mammary skin involving the nipple-areolar complex. She underwent a biopsy of the ulcerative breast tissue, and the histology report did not show a malignant cell. After active foetal lung maturation and induced delivery, a breast reductive surgery with nipple plasty was performed 21 days postpartum. The postoperative course was marked by a period of lymphangitis. The cosmetic and psychological result was satisfactory at 6 months and at 18 months.

**Conclusion:**

Gestational gigantomastia is a benign disease that can simulate carcinomatous mastitis. The breast reductive plasty keeps its place in our context.

## 1. Introduction

Gigantomastia is breast hypertrophy above 1000 grams [1]. This exaggerated mammary hypertrophy could arise during gestational period defined as gravid or gestational gigantomastia [2, 3]. It is a rare condition with unknown etiology [2–6]. Its development during gestational period is in favour of hormonal disequilibrium. It is benign, even though some clinical manifestations call for exclusion of mammary carcinoma [2–4]. Trophic changes are present due to weight pressure of the breast, leading to cutaneous distension. Psychological repercussions are important and are generally described [2]. However, its management is quite controversial in the absence of a recommendation related to the small number of case reports [2–11]. The authors are unanimous on the local care to be brought about the cutaneous ulceration and the inefficiency of the hormonal medical treatment [2, 3]. For this benign disease, the option between radical surgery with immediate reconstruction or differed reconstruction protecting against potential recurrence [7] and mammary reduction surgery exposed to recurrence is a scientific debate [2, 5].

We report a case of bilateral ulcerative gigantomastia in pregnancy to call for the attention of clinicians on this benign condition and to demonstrate the place of a multidisciplinary approach and that of reductive surgery in its management which remains controversial.

## 2. Case

**Figure 1:**
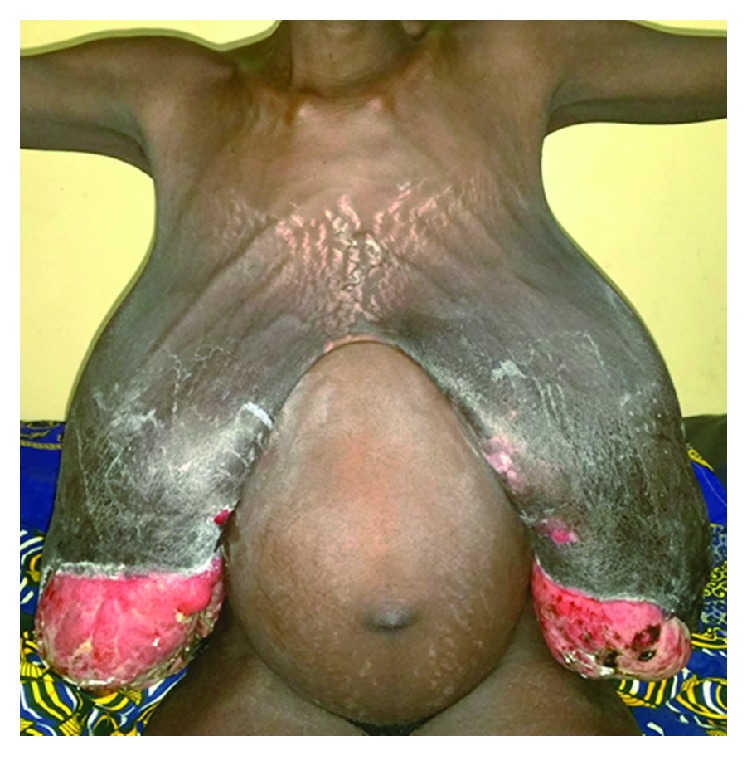
Bilateral giant breasts at 32-week gestation, with collateral venous circulation and trophic changes marked by the necrosis of the distal third of the mammary skin involving the nipple-areolar complex.

A 25-year-old woman gravida 2 para 1 (G2P1) sent by the Maternity Department for an exaggerated bilateral breast enlargement at 32-week gestation. The first pregnancy went on well. There are no similar cases in the family. She did not show signs suggestive of systemic disease including systemic lupus erythematosus. The examination showed bilateral giant breasts with collateral venous circulation and trophic changes marked by the necrosis of the distal third of the mammary skin involving the nipple-areolar complex (Figure 1). The histology of the biopsied ulcerative mammary gland was in favour of a subchronic inflammatory tissue without abscess. The biological search for autoantibodies like ANA, anti-ENA, and anti-dsDNA could not be done because it is not available.

**Figure 2:**
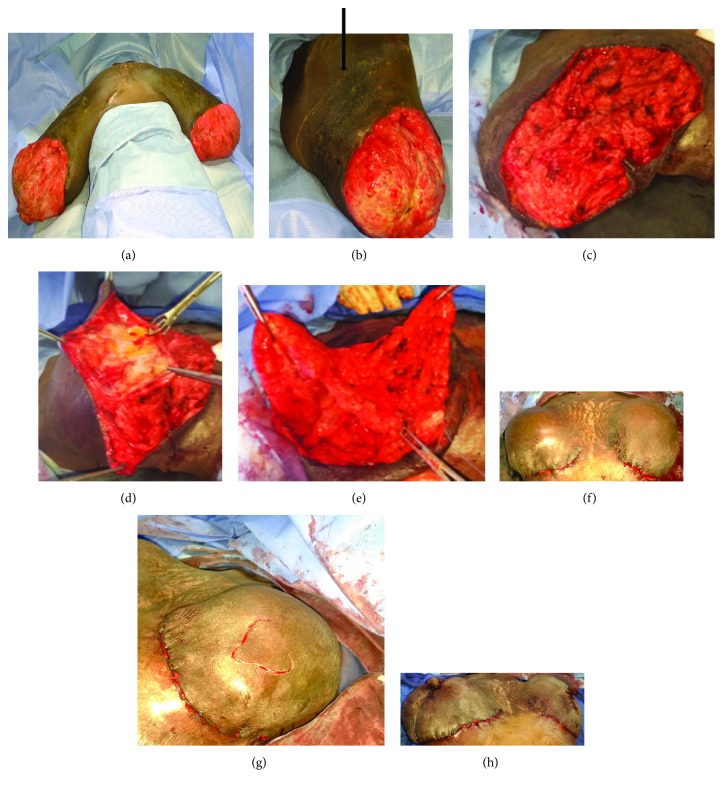
(a) Preoperative installation at D21 postpartum. (b) Arrow showing the incision site for the glandular section. (c) Glandular clean cut taking away the necrotic area and keeping the necessary mammary tissue for the plasty. (d) Skin flap lifting following the crests of Duret. (e) Glandular remodeling after reduction. (f) Cutaneous plasty. (g) Incision of the nipple plasty. (h) Cutaneous and nipple plasty (immediate result).

Through this consultation between obstetricians and surgeons, a normal delivery was conducted after foetal lung maturation. Twenty-one days postpartum, a reductive mammary surgery was performed with nipple plasty (Figures 2(a)–2(h)).

**Figure 3:**
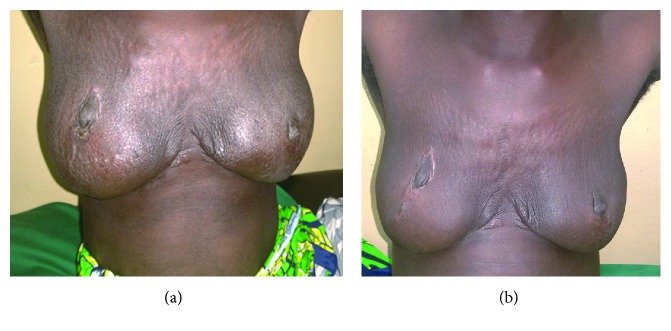
(a) Aesthetic result at 2 months (minimal lymphangitis). (b) Aesthetic result at 18 months.

After a short period of lymphangitis (Figure 3(a)), postoperative follow-up was normal. Cosmetic and psychological result was satisfactory after 18 months (Figure 3(b)); the patient does not want to get pregnant again, but we are following her up regularly to appreciate long-term evolution.

## 3. Discussion

Gestational gigantomastia is defined as one-sided hypertrophic mammary pathology [8] or most often bilateral, associated with rapid and monstrous epithelial hyperplasia during pregnancy [2–7, 10]. Another definition concerns the quantity of the mammary gland, where more than 1500 grams of breast tissues must be removed [1]. In our own case, about 5400 grams of mammary tissues were removed from each breast. It is a rare condition with an incidence rate ranging from 1 in 28,000 to 1 in 100,000 births [2, 3]. A total of 281 cases are reported in the literature [3]. In Burkina, no case has been reported. The risk factors are less illustrated accounting for few cases reported in the literature. The probable associated risk factors are described. The disease occurs in multiparous according to some authors [2, 4, 9], rarely in nulliparous women [11]; in our case study and as reported by other authors [5, 8], it occurs in the second gravidity. White race more involved in the literature, is more an observation than just a rational explanation [12]. We think that cases found in Africa often pass unaware and are not reported in the literature, and even if they are reported, it is in French language and many are not taken into consideration in some meta-analysis. This contributes to the underestimated number of real cases. Though it is rare, it should be known by obstetricians and midwives who are the first to be contacted by pregnant women. In fact, clinical presentation is typically marked by rapid increase in the volume of the breast in the 2nd trimester [2, 3]. According to the size of the breasts, cutaneous dystrophic changes start appearing early leading to ulceration or necrosis of the skin with loss of the nipple-areolar complex like in our case excluding all further functions of the breasts. In addition, there is static disorder associated with instability of the vertebral column due to weight pressure of the huge breasts. Psychological impact is important in the outcome of the pregnancy. It could lead the woman to incriminate the foetal development as the genesis of the disease.

A report incriminates foetal activities as the genesis of the disease [9]. The best knowledge of this benign pathology helps in reassuring the patient and in initiating treatment modalities. It is certainly better to exclude an obvious breast cancer (on one or both sides) or an underlying cancer [3]. This is done by minimal morphological investigations, e.g., ultrasound scan or MRI of the breast, and eventually by an incisional biopsy of the lesions or fine needle aspiration biopsy guided by USS result [8]. Except local treatment to avoid superimposed infections, antalgic, and psychotherapy assistance, specific treatment of this gestational gigantomastia remains controversial [2, 3, 4, 6, 7, 13–15]. Medical treatment with bromocriptine has unanimously proved to be inefficient. Spontaneous resolution has been noted in some minor cases [4, 13]. But this particular case was at the limit of a spontaneous mastectomy with a significant loss of cutaneous substance at the distal 1/3. The nipple-areolar complex was necrotic. After 21 days, there was no tendency to regression. But the weight of the ulcerated breasts caused a permanent embarrassment and repeated dressings. The surgical indication was decided since pregnancy, in front of the extent of skin loss and progressive glandular necrosis. The delay we have given has allowed to decrease the milky surge by physical means and the use of bromocriptine. Of course, there was no breastfeeding, the baby was bottlefed.

Surgery remains the main efficient treatment of this pathology [2, 3, 15]. Surgical modalities seem to find a consensus when it is a gravid gigantomastia in aged multipara who do not desire for more future pregnancy [7]. Reductive surgery is the rule when it is possible to insure good aesthetic result [6, 14], except when the patient prefers bilateral mastectomy with prosthetic or autologous reconstruction [11]; however, in case of a pauciparous young woman who may desire future pregnancy, the choice between reductive surgery and bilateral mastectomy becomes difficult. The last impose an immediate [7] or differed [11] reconstruction according to the local conditions. However, reductive surgery exposes to risk of recurrence in some rare cases that have been described [5]. We opted for this plastic surgery on a patient who rejected bilateral mastectomy despite counseling concerning the risk of recurrence even though she did not desire future pregnancy. Using the inverted T method for mammary reduction with conservation of the nipple-areolar complex, we first proceeded by resection of the mammary gland guided by the average volume desired to remodel. Conservation of NAC was not important since it has been destroyed by the ulceration. Glandular remodeling with a superior pedicle after cutaneous flap lifting was realised to get sufficient curve.

Cutaneous plastic surgery was performed so as to have a single suture in the mammary groove instead of habitual inverted T. Complications are minor apart from the expected lymphangitis that subsided on anti-inflammatory. Aesthetic result was judged well by the patient who did not wish for further repositioning of the nipple and areolar tattoo. Long-term follow-up is ongoing to detect any recurrence.

## 4. Conclusion

Gestational gigantomastia is a benign condition which can simulate a carcinomatous mastitis. Its bilateral characters, clinical aspect, and typical trophic changes in pregnancy are sufficient to make the diagnosis where hormonal impregnation is incriminated. Pluridisciplinary interventions help to optimise the difficult choice between continuing pregnancy and the period of surgical intervention. In our context, breast reduction remains a treatment of choice for gestational gigantomastia.

## References

[1] Chavoin J. P., André A., Meresse T., El Mahrabi H., Grolleau J. L., Chavoin J. P., Amar E., André A., Bodin F. (2012). Plastie mammaire de diminution pour hypertrophie et ptôse. *Chirurgie Plastique et Reconstructive du Sein*.

[2] Boulanger L., SidAhmed-Mezi M., Dhollande A. (2018). How I do…for the surgical management of gestational gigantomastia. *Gynécologie Obstétrique Fertilité & Sénologie*.

[3] Mangla M., Singla D. (2017). Gestational gigantomastia: a systematic review of case reports. *Journal of Mid-life Health*.

[4] Bukhari S. S., Manan H., Khan M. M., Raza S. S. (2018). Resolution of gestational gigantomastia with termination of pregnancy. *Journal of Ayub Medical College Abbottabad-Pakistan*.

[5] Zhou M., Jin M., Wang L., Pan L. J. (2017). Pregnancy-associated gigantomastia recurrence and ectopic breast after reduction mammaplasty: a case report. *Cancer Biomarkers*.

[6] Zingaretti N., Biasio F. D., Riccio M., Nardini N., Mariuzzi L., Parodi P. C. (2017). A case of gestational gigantomastia in a 37-years-old woman associated with elevated ANA: a casual linkage?. *Pan African Medical Journal*.

[7] Nail-Barthelemy R., Burin des Roziers B., Daoud G., Cartier S. (2015). Breast reconstruction for gigantomastia complicating pregnancy. A case report. *Annales de Chirurgie Plastique Esthétique*.

[8] Kuhn-Beck F., Foessel L., Bretz-Grenier M. F., Akladios C. Y., Mathelin C. (2014). Unilateral gigantomastia of pregnancy: Case-report of a giant breast hamartoma. *Gynécologie Obstétrique & Fertilité*.

[9] Modarressi T., Levine M. A., Tchou J., Khan A. N. (2018). Gestational gigantomastia complicated by PTHrP-mediated hypercalcemia. *The Journal of Clinical Endocrinology & Metabolism*.

[10] Dancey A., Khan M., Dawson J., Peart F. (2008). Gigantomastia – a classification and review of the literature. *Journal of Plastic, Reconstructive & Aesthetic Surgery*.

[11] Lapid O. (2013). Breast reconstruction after mastectomy for gestational gigantomastia. *Aesthetic Plastic Surgery*.

[12] Lafreniere R., Temple W., Ketcham A. (1984). Gestational macromastia. *The American Journal of Surgery*.

[13] Dharini, Venkataram T., Raghuprakash S. (2018). Gestational gigantomastia with spontaneous resolution in an Indian woman. *BMJ Case Reports*.

[14] Austin R. E., Lista F., Ahmad J. (2016). Management of recurrent or persistent macromastia. *Clinics in Plastic Surgery*.

[15] Swelstad M. R., Swelstad B. B., Rao V. K., Gutowski K. A. (2006). Management of gestational gigantomastia. *Plastic and Reconstructive Surgery*.

